# Preclinical evidence of reno-protective effect of quercetin on acute kidney injury: a meta-analysis of animal studies

**DOI:** 10.3389/fphar.2023.1310023

**Published:** 2023-12-22

**Authors:** Yi-Fan Zeng, Jing-Yu Li, Xin-Yu Wei, Si-Qing Ma, Qiu-Guo Wang, Zhen Qi, Zhi-Cheng Duan, Ling Tan, Hao Tang

**Affiliations:** ^1^ Department of Cardiovascular Surgery, The Second Xiangya Hospital, Central South University, Changsha, Hunan, China; ^2^ Department of Pharmacy, Xiangya Hospital, Central South University, Changsha, Hunan, China; ^3^ National Clinical Research Center for Geriatric Disorders, Xiangya Hospital, Central South University, Changsha, China; ^4^ Department of Pharmacy, Hunan Chest Hospital, Changsha Medical University, Changsha, China

**Keywords:** quercetin, acute kidney injury, preclinical evidence, meta-analysis, animal

## Abstract

**Objective:** This study evaluated the reno-protective effects of quercetin in animal models of acute kidney injury (AKI).

**Methods:** We conducted a systematic search of literature published before April 2023 in PubMed, Web of Science, and EMBASE databases. Methodological quality was assessed by SYRCLE’s RoB tool. Funnel plot, Egger’s test, and Begg’s test were used to determine publication bias.

**Results:** A total of 19 studies with 288 animals were included in this meta-analysis. The methodology quality scores of the included studies ranged from 4 to 7. The results indicated that quercetin reduced blood urea nitrogen (SMD = −4.78; 95% CI: 6.45, −3.12; *p* < 0.01; I^2^ = 84%) and serum creatinine (SMD: 2.73, 95% CI: 3.66, −1.80; *p* < 0.01; I^2^ = 80%) in AKI models. The result of sensitivity analysis was stable, while the results of funnel plot indicated asymmetric. In addition, we further analyzed inflammatory cytokines, oxidative stress levels, and kidney injury scores, and found that quercetin treatment had antioxidant and anti-inflammatory effects and improved kidney injury scores in animal models of AKI.

**Conclusion:** Quercetin exhibited a promising reno-protective effect in AKI animal models.

**Systematic Review Registration:** PROSPERO (CRD42023433333).

## 1 Introduction

Acute kidney injury (AKI) is a heterogeneous group of diseases in which the renal excretory function decreases rapidly and significantly in a short timeframe (Bellomo et al., 2012; Levey and James, 2017). AKI can be triggered by various factors, including sepsis, shock, cardiac surgery, major non-cardiac surgery, nephrotoxic medications, burns, trauma, contrast agents, and toxins (Kellum et al., 2013). It is accompanied by the accumulation of nitrogenous waste products such as creatinine and urea. Commonly observed complications include volume overload, electrolyte disturbances, uremic syndrome, and drug toxicity. In clinical practice, the diagnosis of AKI is typically based on the guideline from Kidney Disease Improving Global Outcomes (KDIGO) (Kellum et al., 2013), which involves assessing serum creatinine (sCr), estimating glomerular filtration rate, or monitoring urine output. AKI often occurs in patients with underlying acute or chronic diseases, with approximately 20% of hospitalized patients. Among these patients, approximately 10% necessitate renal replacement therapy, and the mortality rate for this subset can be as high as 50% (James et al., 2010; Wonnacott et al., 2014; Wang et al., 2017). Currently, the management of AKI primarily focuses on addressing the underlying causes and preventing associated complications (Kellum et al., 2013). At present, apart from dialysis, there is no effective drug to eliminate AKI (Zuk and Bonventre, 2016; Moore et al., 2018; Li et al., 2022). In cases of severe illness, renal replacement therapy can be employed (Gaudry et al., 2016; Bagshaw and Wald, 2017).

Quercetin is a natural flavonoid compound that is abundant in vegetables, fruits, and medicinal herbs (Chen Y. Q. et al., 2022). Recent studies have found quercetin exhibits diverse biological activities and pharmacological effects, including antioxidant, anti-inflammatory, and anti-allergic properties, inhibits platelet aggregation, and regulates blood pressure and lipid levels (Marunaka et al., 2017; Patel et al., 2018; Hosseini et al., 2021; Di Petrillo et al., 2022). Additionally, quercetin demonstrates promising anticancer effects by inhibiting tumor cell growth, and metastasis, and inducing apoptosis (Reyes-Farias et al., 2019). As a result, quercetin holds significant therapeutic potential in cardiovascular diseases, cancer, and metabolic endocrine disorders.

Recent studies suggested that quercetin may exert protective effects against AKI through various mechanisms. However, due to the diverse etiology of AKI and the variability in animal models used, there is still a significant gap between experimental models and clinical pathology (van der Worp et al., 2010; McGonigle and Ruggeri, 2014; Rui et al., 2022). A possible explanation is that certain animal model does not reflect disease in humans sufficiently. Moreover, negative studies may be more difficult to publish than neutral clinical trials. However, how should we improve the reproducibility and translation of animal research? The first and most important recommendation of Rainer Spanagel’s list of 10 recommendations for improving reproducibility and translation is to perform a preclinical meta-analysis (Spanagel, 2022).

This preclinical meta-analysis aims to assess the reno-protective effects of quercetin in AKI animal models and lay the groundwork for future clinical investigations. We believe our research may provide an in-depth vision for subsequent studies.

## 2 Methods

We have pre-registered this meta-analysis in PROSPERO (identifier: CRD42023433333). We used the Covidence platform to conduct the process of study selection, data extraction, and quality assessment. Therefore, the way to resolve disagreements was the same in the above process. For example, in the process of Data Extraction, standard data extraction forms were first created, and then two independent reviewers extracted data from included studies. After they finished the process of data extraction, a third independent reviewer checked and merged the data. Group discussion would resolve any disagreements that arose.

### 2.1 Search strategy

We systematic search of literature published to April 2023 in Web of Science, PubMed, and Embase. No language restrictions were set. “acute kidney injury”, “acute kidney failure”, “acute renal insufficiency”, “kidney ischemia/reperfusion injury”, “renal ischemia/reperfusion injury”, “kidney ischemia/reperfusion”, “quercetin”, “dikvertin”, and “3,3′,4′,5,7-pentahydroxyflavone” were used as index keywords.

### 2.2 Study selection

The included criteria were as follows: 1) AKI models: drug-induced (DKI), ischemia/reperfusion-induced, and LPS-induced AKI animal models; 2) Treatment: the only intervention was quercetin; 3) Control group: no treatment or placebo fluid control; and 4) Data: detailed data of primary and/or secondary outcomes contained (see in **Data Extraction**). Exclusion Criteria were listed as follows: 1) no detailed data, 2) *in vitro* cell study or no animal model, 3) no control group was set, 4) quercetin is not the only intervention, 5) *in vitro* cell studies, 6) article types: review, case reports, conference abstracts, and meta-analysis.

### 2.3 Data extraction

Information extracted includes as follows: 1) Author information: author, year, and country; 2) Animal information: species, weight, sex, and sample size; 3) Animal model: model methods; 4) Drug: administration method, dosage, and duration; 5) Outcomes: extract the mean and standard deviation (SD) data of the primary and secondary outcome in detail. Engauge Digitizer (version 12.1) is used for data extraction when the data is included in the Figures.

Primary outcomes include blood urea nitrogen (BUN) and sCr. Secondary outcomes include superoxide dismutase (SOD), malondialdehyde (MDA), catalase (CAT), glutathione (GSH), interleukin-1β (IL-1β), tumor necrosis factor-α (TNF-α), interleukin-6 (IL-6), and kidney injury score (KIS). When different doses or duration of quercetin were administrated, data with the highest dose and longest duration would be extracted.

### 2.4 Quality assessment

Two independent authors conducted the quality assessment of the included studies by Risk of Bias (RoB) tools for animal intervention studies (Hooijmans et al., 2014). The ten entries of SYRCLE’s RoB tools include Sequence generation, Baseline characteristics, Allocation concealment, Random housing, Blinding investigators, Random outcome assessment, Blinding outcome assessor, Incomplete outcome data, Selective outcome reporting, and Other sources of bias.

### 2.5 Statistical analysis

Since this was a meta-analysis of preclinical animals, all pooled estimates were performed using a random effects model. Standard mean difference (SMD) was used as a calculation for the pooled estimates due to different animal species and animal models. Heterogeneity assessment was conducted by the Cochran Q test and the I^2^ statistics. When more than 10 studies were included, subgroup analysis, sensitivity analysis, and publication bias analysis were conducted. Leave-one-out method was used to conduct sensitivity analysis, and the research would be removed when the 95% CI of the pooled estimate after a study is excluded, changes significantly, and crosses the nullity line. In this meta-analysis, no study was removed at all. When the *p*-value between subgroups was less than 0.05 or I^2^ decreased significantly, we thought this group may be an important source of heterogeneity. Publication bias was evaluated by funnel plot, Begg’s test, and Egger’s test. If the funnel plot is asymmetrical or the *p*-value of Begg’s and Egger’s tests is less than 0.05, it is considered that publication bias may exist, and *vice versa*. The difference was considered statistically significant when the *p*-value was less than 0.05. All analysis above were analyzed by R (Version 4.2.2) with packages “meta” and “metafor”.

## 3 Results

### 3.1 Study selection

**FIGURE 1:**
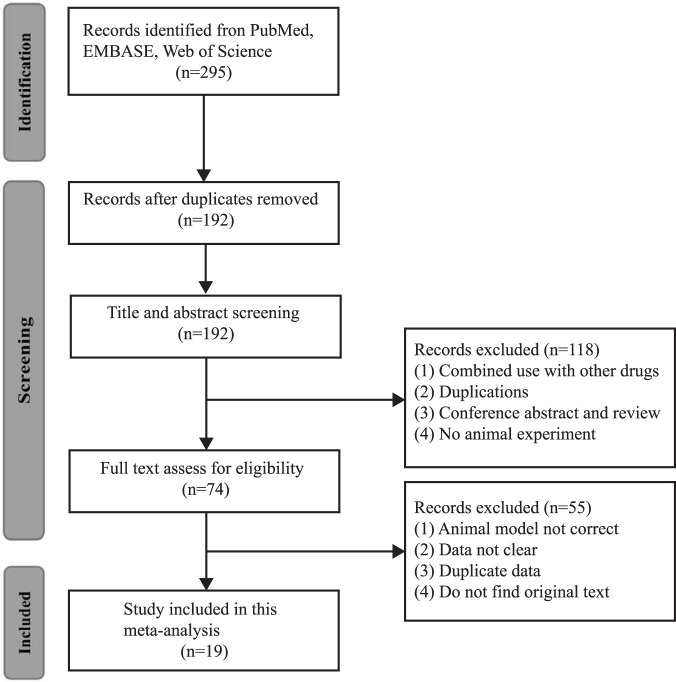
Flow diagram of database searches and study selection.

A total of 295 studies were involved in the primary retrieval, of which 103 were reduplicated articles. Then, 118 articles were excluded because they were 1) combined use with other drugs, 2) duplications, 3) conference abstracts and reviews, and 4) no animal experiment. After reading the full-text articles, 55 studies were excluded due to 1) the animal model not being correct, 2) data not being clear, 3) duplicate data, and 4) the original text not being available. Finally, 19 studies were eligible in which 288 animals were included (Chander et al., 2005; Kusumoto et al., 2011; Sanchez-Gonzalez et al., 2011; Nabavi et al., 2012; Chen et al., 2014; Saito et al., 2014; Li et al., 2016; Gholampour and Sadidi, 2018; Shu et al., 2018; Lucida et al., 2019; Tan et al., 2019; Tan et al., 2020; El-Sayed et al., 2021; Goncalves et al., 2021; Rahdar et al., 2021; Wang et al., 2020; Ali et al., 2022; Vazquez-Atanacio et al., 2022; Yilmaz et al., 2022) (Figure 1).

### 3.2 Baseline characteristics of included studies

The 19 studies included in this meta-analysis were published between 2005 and 2022 and the main characteristics of these studies were summarized in Table 1. Six studies were conducted in China, 3 studies in Iran, 2 studies in Japan, and 1 study each in Brazil, Egypt, India, Indonesia, Iraq, Mexico, Spain, or Turkey. AKI was induced by drugs in 12 studies, ischemia-reperfusion (I/R) in 5 studies, and lipopolysaccharides (LPS) in 2 studies. To establish AKI animal model, 5 studies used Wistar rats, 4 studies used C57BL/6 mice, 4 studies used SD rats, 3 studies used albino Wistar rats, 1 study each used Fischer F344 rats, BALB/c mice, and white mice. In addition, except for 1 study that used female animals all of the others were male animals. Quercetin was administered intragastric in 11 studies, intraperitoneal in 8 studies. The treatment duration of quercetin ranged from 30 min to 12 weeks.

**TABLE 1:** Baseline characteristics of included studies.

Author	Year	Country	Species (weight)	Sex	N (Control)	N (quercetin)	Model method	Drug delivery method	Duration	Indicators
Ali	2022	Iraq	albino Wistar rats (150–230 g)	Male	8	8	DKI (5-fluorouracil)	intragastric (80 mg/kg/d)	14 days	BUN, sCr
Buys-Goncalves	2021	Brasil	Wistar rats	Male	10	10	I/R induced	intragastric (50 mg/kg/d)	6 days	BUN, sCr
Chander	2005	India	Wistar rats (150–200 g)	Male	8	8	DKI (glycerol (50%, v/v))	intraperitoneal (2 mg/kg/d)	30 min	BUN, sCr, CAT, SOD, GSH
Chen	2014	China	C57BL/6J mice	Male	4	4	I/R induced	intraperitoneal (10 mg/kg/d)	1 day	sCr
El-Sayed	2021	Egypt	Wistar rats (200–250 g)	Male	6	6	I/R induced	intragastric (50 mg/kg/d)	19 h	BUN, sCr, CAT, SOD, GSH
Gholampour	2018	Iran	SD rats (250–300 g)	Male	7	7	I/R induced	intraperitoneal (10 mg/kg/d)	1 day	BUN, sCr, CAT, MDA
Kusumoto	2011	Japan	SD rats	Male	5	5	DKI (cisplatin)	intragastric (50 mg/kg/d)	3 days	BUN, sCr, MDA
Li	2016	China	SD rats (170–190 g)	Male	6	6	DKI (cisplatin) in DMH-induced colon cancer model	intraperitoneal (50 mg/kg/d)	7 days	BUN, sCr
Lucida	2019	Indonesia	white mice (20–30 g)	Male	7	7	DKI (Gentamycin)	intragastric (50 mg/kg/d)	7 days	sCr
Nabavi	2012	Iran	Wistar rats (200–250 g)	Male	10	10	DKI (NaF)	intraperitoneal (20 mg/kg/d)	7 days	BUN, sCr, CAT, SOD, GSH
Rahdar	2021	Iran	Wistar rats (200–250 g)	Male	7	7	DKI (Gentamycin)	intragastric (50 mg/kg/d)	10 days	BUN, sCr, CAT, MDA, SOD, GSH
Saito	2014	Japan	SD rats	Male	16	16	I/R induced	intragastric (50 mg/kg/d)	2 days	BUN, sCr
Sanchez-Gonzalez	2011	Spain	Fischer F344 rats (∼200 g)	Male	5	5	DKI (cisplatin) in breast adenocarcinoma animal model	intraperitoneal (50 mg/kg/d)	6 days	BUN, sCr, MDA, TNF-α, KIS
Shu	2018	China	C57BL/6J mice (18–22 g)	Male	6	6	LPS induced	intraperitoneal (40 mg/kg/d)	3 days	BUN, TNF-α, IL-1β, IL-6, KIS
Tan	2020	China	C57BL/6 mice (22–25 g)	Male	10	10	DKI (cisplatin)	intraperitoneal (100 mg/kg/d)	3 days	BUN, sCr, TNF-α, IL-1β, IL-6, KIS
Tan	2019	China	BALB/c mice (22–25 g)	Male	10	10	LPS induced	intragastric (50 mg/kg/d)	3 days	BUN, sCr, TNF-α, IL-1β, IL-6, KIS
Vazquez-Atanacio	2022	Mexico	albino Wistar rats (250–300 g)	Male	5	5	DKI (thioacetamide, TAA)	intragastric (50 mg/kg/d)	5 days	BUN, sCr
Wang	2020	China	C57BL/6J mice (20–22 g)	Male	6	6	DKI (folic acid); I/R	intragastric (25 mg/kg/d)	24 h	BUN, sCr, MDA, GSH, TNF-α, IL-1β, IL-6, KIS
Yilmaz	2022	Turkey	albino Wistar rats (220–250 g)	Female	8	8	DKI (meglumine sodium diatrizoate)	intragastric (50 mg/kg/d)	4 days	BUN, sCr, KIS

Drug-induced kidney injury, DKI; lipopolysaccharide, LPS; 1,2-dimethyl hydrazine, DMH; blood urea nitrogen, BUN; serum creatinine, sCr; superoxide dismutase, SOD; catalase, CAT; malondialdehyde, MDA; glutathione, GSH; interleukin-1β, IL-1β; interleukin-6, IL-6; tumor necrosis factor-α, TNF-α; kidney injury scores, KIS.

The quality assessment of the included studies scored from 4 to 7 (Table 2). The verified molecular mechanism of which reno-protective effect of quercetin in AKI was summarized in Table 3.

**TABLE 2:** Quality assessment of included studies by the SYRCLE’s RoB tool.

Author	Year	A	B	C	D	E	F	G	H	I	J	Total
Ali	2022		★	★	★		★			★		5
Buys-Goncalves	2021		★	★	★		★		★	★		6
Chander	2005		★	★			★			★		4
Chen	2014		★	★			★			★		4
El-Sayed	2021		★	★	★		★		★	★		6
Gholampour	2018		★	★	★		★			★		5
Kusumoto	2011		★	★			★			★		4
Li	2016		★	★	★		★		★	★		6
Lucida	2019		★	★	★		★			★		5
Nabavi	2012		★	★	★		★		★	★		6
Rahdar	2021		★	★	★		★		★	★	★	7
Saito	2014		★	★			★			★		4
Sanchez-Gonzalez	2011		★	★	★		★		★	★		6
Shu	2018		★	★	★		★		★	★	★	6
Tan	2020		★	★			★			★		4
Tan	2019		★	★	★		★			★		5
Vazquez-Atanacio	2022		★	★			★		★	★		5
Wang	2020		★	★			★		★	★		5
Yilmaz	2022		★	★			★			★		4

A, sequence generation; B, baseline characteristics; C, allocation concealment; D, random housing; E, blinding investigators; F, random outcome assessment; G, blinding outcome assessor; H, incomplete outcome data; I, selective outcome reporting; J, other sources of bias.

**TABLE 3:** The molecular and cellular mechanisms underlying the reno-protective effect of quercetin treatment in AKI.

Author	Year	Proposed mechanisms
Ali	2022	Decrease the levels of KIM-1, NGAL, and cys-C, and the novel inflammatory markers of kidney injury like NLP, MLR, and PLR, as well as decrease uric acid and restore the TAOC
Buys-Goncalves	2021	NA
Chander	2005	Anti-oxidative stress
Chen	2014	Activate AMPK-regulated autophagy signaling pathway
El-Sayed	2021	Regulate Rho-kinase pathway and the production of H2S
Gholampour	2018	Reduction of lipid peroxidation and augmentation of antioxidant systems
Kusumoto	2011	Reduce accumulation of indoxyl sulfate
Li	2016	NA
Lucida	2019	NA
Nabavi	2012	NA
Rahdar	2021	Antioxidant activity
Saito	2014	Inhibit hepatic SULT activity
Sanchez-Gonzalez	2011	Prevent the nephrotoxic effect of cisplatin without affecting its anti-tumour activity
Shu	2018	Blockade of CD38, inhibit macrophage activation, and suppress NF-κB signaling pathway
Tan	2020	Inhibit Mincle/Syk/NF‐κB signaling maintained macrophage inflammation
Tan	2019	Suppress TLR4/NF‐κB pathway
Vazquez-Atanacio	2022	NA
Wang	2020	Inhibit ferroptosis by inhibitting the expression of ATF3
Yilmaz	2022	NA

Neutrophil/Lymphocyte, NLR; Monocyte/Lymphocyte, MLR; Platelets/Lymphocyte Ratio, PLR; sulfotransferase, SULT; Macrophage‐inducible C‐type lectin, Mincle; Activation transcription factor 3, ATF3; nuclear factor-κB, NF‐κB; AMP-activated protein kinase, AMPK; total antioxidant capacity, TOAC; neutrophil gelatinase-associated lipocalin, NGAL; kidney damage molecule, KIM-1.

### 3.3 Outcome measures

#### 3.3.1 Blood urea nitrogen

**FIGURE 2:**
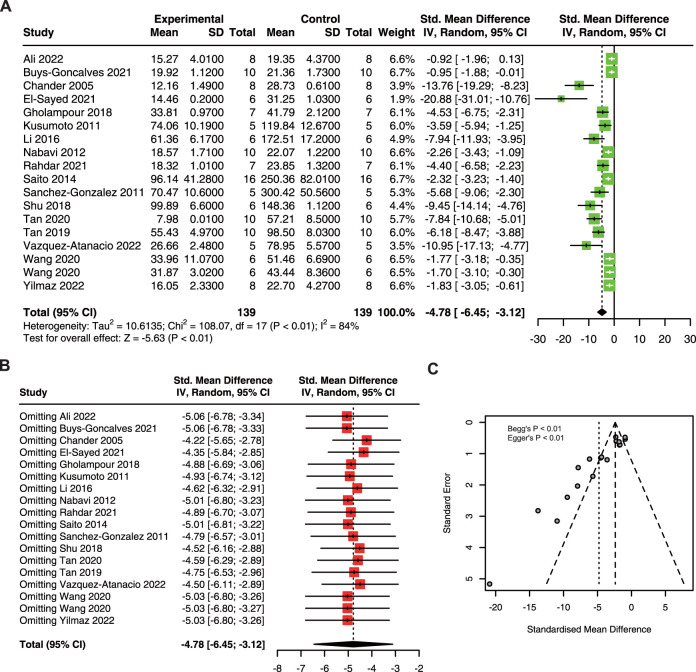
The protective effect of quercetin on BUN in AKI animals. **(A)** Forest plot showing the protective effect of quercetin on BUN in preclinical animal model of AKI. **(B)** Funnel plot assessing publication bias of BUN in included studies. **(C)** Sensitivity analysis of BUN. BUN, blood urea nitrogen. AKI, acute kidney injury.

This meta-analysis included 18 research with 278 animals (Chander et al., 2005; Kusumoto et al., 2011; Sanchez-Gonzalez et al., 2011; Nabavi et al., 2012; Saito et al., 2014; Li et al., 2016; Gholampour and Sadidi, 2018; Shu et al., 2018; Tan et al., 2019; Tan et al., 2020; El-Sayed et al., 2021; Goncalves et al., 2021; Rahdar et al., 2021; Wang et al., 2020; Ali et al., 2022; Vazquez-Atanacio et al., 2022; Yilmaz et al., 2022). The result showed that quercetin administration obviously decreased BUN (SMD = −4.78; 95% CI: 6.45, −3.12; *p* < 0.01; I^2^ = 84%) in AKI animals (Figure 2A).

Due to the high heterogeneity in this meta-analysis, we performed sensitivity analysis and the results suggested that ignoring a single study had no remarkable effect on the pooled estimates of BUN (Figure 2B). The asymmetry of the funnel plot indicated the existence of publication bias (Figure 2C), and the *p*-value of Begg’s and Egger’s tests were lower than 0.01.

To explore the source of heterogeneity, we performed subgroup analysis using species, animal models, dose, drug delivery methods, and duration. The results exhibited that the difference between intragastric and intraperitoneal was statistically significant (Table 4). Besides, the heterogeneity of LPS-induced AKI models was minimal.

**TABLE 4:** Subgroup analysis of pooled estimates of BUN.

Subgroup	No. of studies	SMD	95% CI	*p*-Value between subgroups	Heterogeneity within subgroups	
					I^2^ (%)	*p*-Value
**Species**				0.68		
Wistar rats	8	−5.84	−10.08, −1.60		87	<0.01
SD rats	4	−4.02	−5.97, −2.07		70	0.02
Fischer F344 rats	1	−5.68	−9.06, −2.30		—	—
C57BL/6 mice	4	−4.80	−8.64, −0.95		88	<0.01
BALB/c mice	1	−6.18	−8.47, −3.88		—	—
**Model**				0.40		
DKI	11	−4.84	−6.95, −2.72		84	<0.01
I/R	5	−4.38	−9.01, 0.26		83	<0.01
LPS	2	−7.15	−10.08, −4.22		34	0.22
**Dose**				0.81		
≤20 mg/kg	3	−6.37	−12.80, 0.07		89	<0.01
20–40 mg/kg	3	−3.80	−8.27, 0.67		80	<0.01
>40 mg/kg	12	−4.76	−6.72, −2.80		86	<0.01
**Drug Delivery**				0.02		
Intraperitoneal	7	−6.76	−9.37, −4.16		84	<0.01
Intragastric	11	−3.10	−4.50, −1.70		80	<0.01
**Duration**				0.35		
≤1day	5	−7.46	−14.03, −0.89		88	<0.01
1day-1week	11	−4.70	−6.53, −2.87		84	<0.01
>1week	2	−2.53	−5.93, 0.88		88	<0.01

#### 3.3.2 Serum creatinine

**FIGURE 3:**
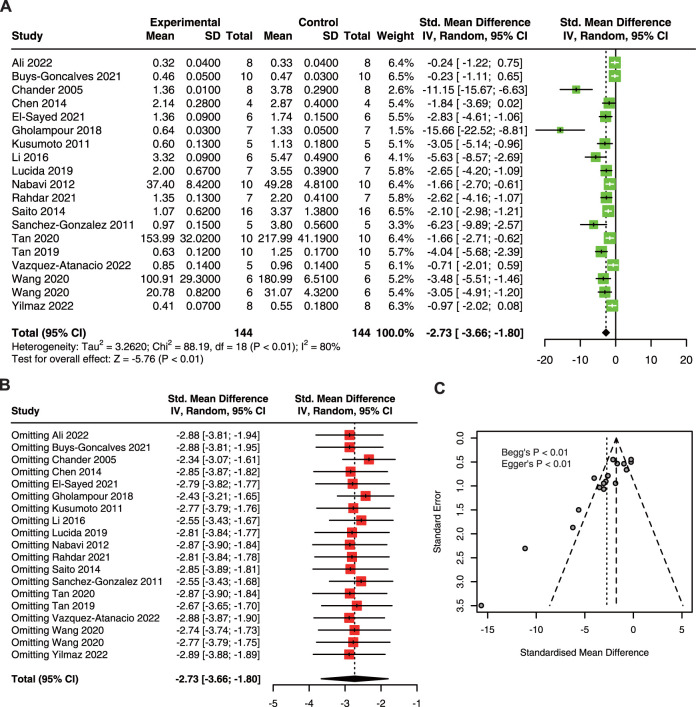
The protective effect of quercetin on sCr in AKI animals. **(A)** Forest plot displaying the protective effect of quercetin on sCr in preclinical animal model of AKI. **(B)** Funnel plot assessing publication bias of sCr in included studies. **(C)** Sensitivity analysis of sCr. sCr, serum creatinine; AKI, acute kidney injury.

As shown in Figure 3A, quercetin pre-treatment significantly decreased the sCr in AKI (SMD: 2.73, 95% CI: 3.66, −1.80; *p* < 0.01; I^2^ = 80%) (Chander et al., 2005; Kusumoto et al., 2011; Sanchez-Gonzalez et al., 2011; Nabavi et al., 2012; Chen et al., 2014; Saito et al., 2014; Li et al., 2016; Gholampour and Sadidi, 2018; Lucida et al., 2019; Tan et al., 2019; Tan et al., 2020; El-Sayed et al., 2021; Goncalves et al., 2021; Rahdar et al., 2021; Wang et al., 2020; Ali et al., 2022; Vazquez-Atanacio et al., 2022; Yilmaz et al., 2022). Furthermore, sensitivity analysis results showed no variation in the pooled estimate of sCr, suggesting that quercetin has a robust effect in reducing sCr in AKI animals (Figure 3B). However, an asymmetrical funnel plot with Begg’s test (*p* < 0.01) and Egger’s test (*p* < 0.01) reasonably indicated publication bias (Figure 3C).

Subgroup analysis showed that there is no significant difference between subgroups (Table 5). However, the heterogeneity was minimal in the dose of 20–40 mg/kg/d.

**TABLE 5:** Subgroup analysis of pooled estimates of sCr.

Subgroup	No. of studies	SMD	95% CI	*p*-Value between subgroups	Heterogeneity within subgroups	
					I^2^ (%)	*p*-Value
**Species**				0.55		
Rats	13	−3.25	−5.02, −1.48		83	<0.01
Mice	6	−2.66	−3.48, −1.83		34	0.18
**Model**				0.46		
DKI	12	−2.73	−3.98, −1.49		78	<0.01
I/R	6	−3.18	−5.90, −0.46		84	<0.01
LPS	1	−4.04	−5.68, −2.39		—	—
**Dose**				0.16		
≤20 mg/kg	4	−7.00	−13.60, −0.39		90	<0.01
20–40 mg/kg	2	−3.25	−4.62, −1.88		0	0.76
>40 mg/kg	13	−2.14	−2.98, −1.30		76	<0.01
**Drug Delivery**				0.05		
Intraperitoneal	7	−5.55	−9.05, −2.06		86	<0.01
Intragastric	12	−1.99	−2.76, −1.23		74	<0.01
**Duration**				0.18		
≤1day	6	−5.60	−9.47, −1.73		81	<0.01
1day-1week	11	−2.18	−3.08, −1.29		74	<0.01
>1week	2	−1.35	−3.68, 0.98		85	0.01

#### 3.3.3 Oxidative stress levels

To explore the antioxidant effect of quercetin treatment on AKI animals. Three studies with 50 animals for SOD, 4 studies with 64 animals for CAT, 6 studies with 72 animals for MDA, and 5 studies with 74 animals for GSH were included.

**FIGURE 4:**
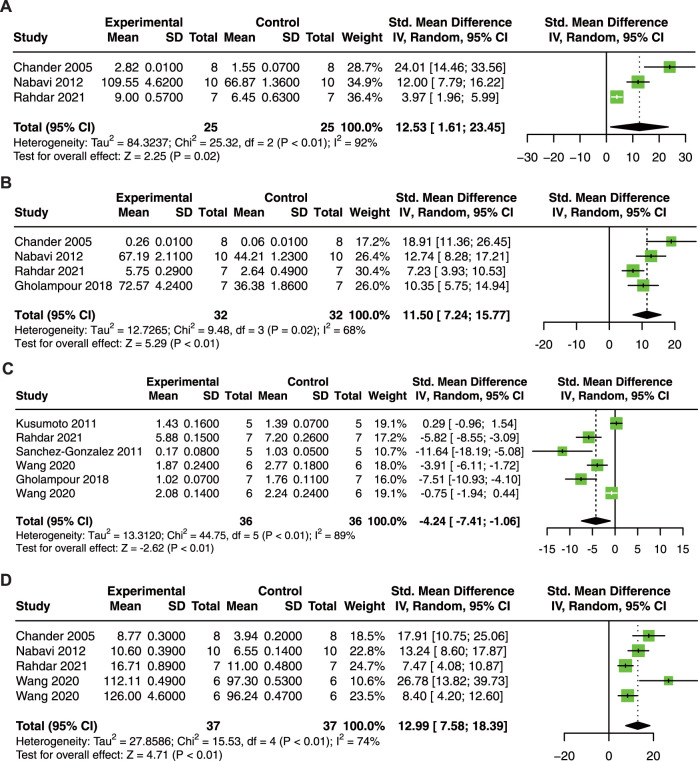
Forest plot showing reno-protective effects of quercetin on SOD **(A)**, CAT **(B)**, MDA **(C)**, and GSH **(D)** in the AKI animal model. SOD, superoxide dismutase; CAT, catalase; MDA, malondialdehyde; GSH, glutathione.

The results showed that quercetin treatment significantly increased SOD (SMD = 12.53; 95% CI: 1.61, 23.45; *p* < 0.01; I^2^ = 92%; Figure 4A) (Chander et al., 2005; Nabavi et al., 2012; Rahdar et al., 2021), CAT (SMD = 11.50; 95% CI: 7.24, 15.77; *p* = 0.02; I^2^ = 68%; Figure 4B) (Chander et al., 2005; Nabavi et al., 2012; Gholampour and Sadidi, 2018; Rahdar et al., 2021), and GSH (SMD = 12.99; 95% CI: 7.58, 18.39; *p* < 0.01; I^2^ = 74%; Figure 4D) (Chander et al., 2005; Kusumoto et al., 2011; Sanchez-Gonzalez et al., 2011; Gholampour and Sadidi, 2018; Rahdar et al., 2021; Wang et al., 2020); and decreased in MDA (SMD = −4.24; 95% CI: 7.41, −1.06; *p* < 0.01; I^2^ = 89%; Figure 4C) (Kusumoto et al., 2011; Sanchez-Gonzalez et al., 2011; Gholampour and Sadidi, 2018; Rahdar et al., 2021; Wang et al., 2020) in animal models of AKI.

#### 3.3.4 Inflammation cytokine

In terms of inflammatory cytokines, 5 studies with 74 animals for TNF-α, and 5 studies with 76 animals for IL-1β and IL-6 were included in the meta-analysis.

**FIGURE 5:**
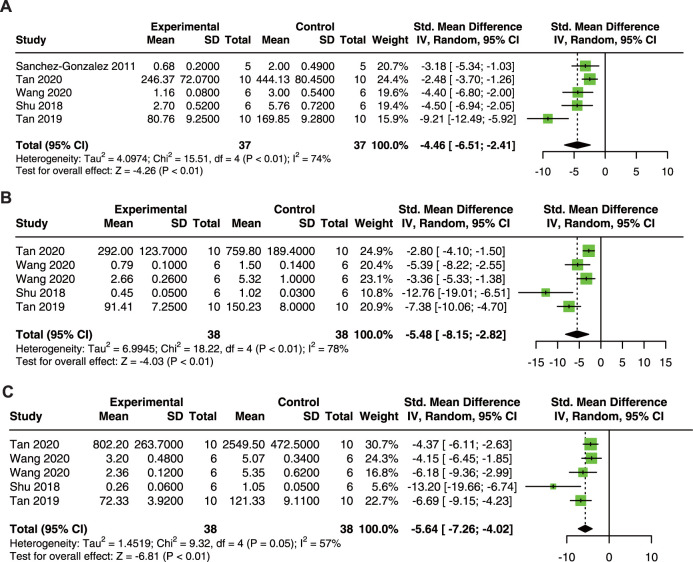
Pooled estimated of TNF-α **(A)**, IL-1β **(B)**, and IL-6 **(C)** after quercetin treatment on AKI animal model. IL-1β, interleukin-1β; IL-6, interleukin-6; TNF-α, tumor necrosis factor-α.

Our results revealed that quercetin administration reduce serum TNF-α (SMD = −4.46; 95% CI: 6.51, −2.41; *p* < 0.01; I^2^ = 74%; Figure 5A) (Sanchez-Gonzalez et al., 2011; Shu et al., 2018; Tan et al., 2019; Tan et al., 2020; Wang et al., 2020), IL-1β (SMD = −5.48; 95% CI: 8.15, −2.82; *p* < 0.01; I^2^ = 78%; Figure 5B) (Shu et al., 2018; Tan et al., 2019; Tan et al., 2020; Wang et al., 2020), and IL-6 (SMD = −5.64; 95% CI: 7.26, −4.02; *p* = 0.05; I^2^ = 57%; Figure 5C) (Shu et al., 2018; Tan et al., 2019; Tan et al., 2020; Wang et al., 2020) in AKI animals. These results indicate that quercetin exerts promising anti-inflammatory effects in AKI.

#### 3.3.5 Kidney injury score

**FIGURE 6:**
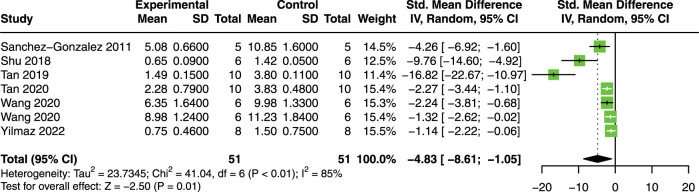
Pooled estimated of kidney injury scores after quercetin treatment on AKI animal model.

Seven studies with 102 animals were included to analyze the effect of quercetin on KIS (Sanchez-Gonzalez et al., 2011; Shu et al., 2018; Tan et al., 2019; Tan et al., 2020; Wang et al., 2020; Yilmaz et al., 2022). Compared with control animals, the results showed that quercetin significantly decreased KIS (SMD = −4.83; 95% CI: 8.61, −1.05; *p* < 0.01; I^2^ = 85%; Figure 6) in AKI animals.

## 4 Discussion

### 4.1 Summary of evidence

This meta-analysis included 19 studies with 288 animals and found that quercetin protects renal function by reducing BUN and sCr levels, decreasing oxidative stress and inflammatory response levels, and reducing KIS.

In subgroup analysis, our results showed no statistically significant was found between the different groups of doses. However, the lowest SMD was found at a dose of ≤20 mg/kg in both sCr and BUN, which revealed that a low dose of quercetin may be considered to be the optimal dose. Moreover, there was no statistically significant found between the different groups of animal models (DKI, I/R, and LPS). Therefore, we thought that quercetin exhibits reno-protective effects in both DKI, I/R, and LPS-induced AKI.

Despite the significant and positive results obtained, further exploration is still required to validate the findings due to the potential high heterogeneity and publication bias included in this meta-analysis.

### 4.2 Molecular mechanisms

AKI can be caused by pre-renal, intrinsic renal, and post-renal factors (Gonsalez et al., 2019), which result in pathological changes such as tubular injury, vascular damage, inflammatory response, cell apoptosis/necrosis, and renal interstitial fibrosis (Jha et al., 2016; Rabb et al., 2016; Dellepiane et al., 2020; Hosohata et al., 2021; Scholz et al., 2021; Li et al., 2022). Quercetin has been shown to exert renal protective effects in various AKI models, preventing the elevation of BUN and sCr, and the decline in eGFR (Kusumoto et al., 2011; Alasmari, 2021; Chen Y. Q. et al., 2022). Additionally, literature reports have demonstrated that quercetin can protect the normal structural integrity of the kidney and reverse histopathological changes in the kidney (Shoskes, 1998; Shu et al., 2018; Han and Lee, 2019; Alasmari, 2021; El-Sayed et al., 2021; Ali et al., 2022). We have summarized the potential mechanisms underlying the renal protective effects of quercetin as follows.

#### 4.2.1 Antioxidants

The accumulation of reactive oxygen species (ROS) or a decrease in antioxidant substances (SOD, GSH, CAT, *etc.*) in the body leads to an oxidative stress state. This imbalance between oxidation and antioxidation is considered one of the crucial factors contributing to the occurrence and progression of AKI.

As one of the polyphenolic compounds, quercetin possesses a phenolic ring structure that facilitates electron donation and hydrogen atom transfer to free radicals (Dabulici et al., 2020). This property allows quercetin to effectively scavenge free radicals, exhibiting excellent antioxidant characteristics (Chander et al., 2005). Dates from animal models of AKI have shown that quercetin increases antioxidant (GSH, vitamin C, vitamin E, *etc.*) levels (Kahraman et al., 2003; Renugadevi and Prabu, 2010; Kinaci et al., 2012; Nabavi et al., 2012; Sanchez-Gonzalez et al., 2017), strengthens the enzymes activity of GSH, CAT, SOD (Inal et al., 2002; Kahraman et al., 2003; Singh et al., 2004; Renugadevi and Prabu, 2010; Yao et al., 2011; Nabavi et al., 2012; Erboga et al., 2015; Elbe et al., 2016; Sanchez-Gonzalez et al., 2017; Yuksel et al., 2017; Gholampour and Sadidi, 2018), decreases the concentration of oxidants (e.g., MDA) (Kinaci et al., 2012; Erboga et al., 2015; Elbe et al., 2016; Yuksel et al., 2017; Gholampour and Sadidi, 2018), and reduces renal lipid peroxidation (Singh et al., 2004; Renugadevi and Prabu, 2010; Chaudhary et al., 2015; Gholampour and Sadidi, 2018; Morsi et al., 2022).

#### 4.2.2 Anti-inflammation

Different etiological factors contribute to renal damage, leading to the infiltration of inflammatory cells, activation of inflammatory cytokines, accumulation of macrophages, and the release of danger-associated molecular patterns (DAMPs) (Cai et al., 2022). In AKI, markers of renal function are significantly elevated, along with elevated levels of inflammatory mediators including NF-κB, TNF-α, IL-6, and myeloperoxidase (MPO), indicating that inflammatory are involved in the pathogenesis of kidney injury (Zuk and Bonventre, 2016; Ansari et al., 2017).

Quercetin interacts with multiple targets involved in the inflammatory response of AKI, thereby suppressing the inflammatory process. Quercetin inhibits the production of TNF-α, IL-1β, and IL-6, and reduces MPO activity in LPS-induced AKI models, Sepsis-induced AKI models, or I/R-induced AKI models (Kahraman et al., 2003; Liu et al., 2005; Kinaci et al., 2012; Tan et al., 2020). In addition, quercetin exerts its anti-inflammatory effects in AKI models by inhibiting the expression of TLR4, MyD88, and TRAF-6 in rat kidneys, and suppresses the NF-κB signaling pathway (Kinaci et al., 2012; Elbe et al., 2016; Shu et al., 2018; Tan et al., 2019). Furthermore, quercetin alleviates the inflammatory response in AKI models by modulating macrophage polarization and reducing macrophage infiltration (Nakamura and Omura, 2008; Lu et al., 2018; Shu et al., 2018; Tan et al., 2020).

#### 4.2.3 Cell death

Various forms of cell death pathways may be involved in the pathophysiological processes of AKI, including apoptosis, necroptosis, ferroptosis, and pyroptosis (Ni et al., 2022; Sanz et al., 2023). Quercetin attenuates cell injury and apoptosis in contrast-induced AKI models by inhibiting HIF-1α on the lncRNA NEAT1/HMGB1 signaling pathway (Luo et al., 2022). In indomethacin-induced AKI, quercetin protects HEK293 cells against cell apoptosis by reducing ROS production, increasing mitochondrial membrane potential, and down-regulating apoptotic expression of the caspase-3 and caspase-9 signals (Chen et al., 2021). Moreover, quercetin significantly suppressed the nephrotoxic effect of cisplatin by inhibiting tubular necrosis/apoptosis and the activity of the apoptosis executioner caspase-3 (Sanchez-Gonzalez et al., 2011). Wang et al., identified quercetin as a ferroptosis inhibitor, downregulated ATF3, and inhibited the chemotaxis of macrophages (Wang et al., 2020). In addition, recent findings demonstrated that quercetin improved renal cell pyroptosis via the IL33/ST2 pathway *in vitro* (Chen H. Y. et al., 2022). Overall quercetin can reduce the extent of kidney injury via inhibition of cell death pathways.

#### 4.2.4 Autophagy

Autophagy degrades and recycles damaged organelles and macromolecules to maintain cellular homeostasis. In AKI models, impaired autophagy leads to increased levels of p62 and oxidative stress, resulting in exacerbated damage to renal tubules and renal function (Kaushal and Shah, 2016). In addition, suppression of autophagy may also enhance apoptosis (Periyasamy-Thandavan et al., 2008; Jiang et al., 2010). Numerous studies have indicated that quercetin plays a role in modulating autophagy (Wu et al., 2017; Han et al., 2021). Chen et al., reveal that quercetin activates an AMPK-regulated autophagy signaling pathway and offers a protective effect in I/R-induced AKI (Chen et al., 2014).

#### 4.2.5 Mitochondrial protection

Mitochondrial dysfunction plays a crucial role in the pathogenesis of AKI. In AKI, damaged mitochondria accumulate within cells, leading to increased reactive oxygen species (ROS) production, oxidative stress, and even triggering mitochondria-dependent apoptosis (Duann and Lin, 2017; Jiang et al., 2020). Recent studies have proposed that quercetin can protect mitochondria, reduce mitochondrial apoptosis, and exert a reno-protective effect (Shirani et al., 2019; Peng et al., 2020).

### 4.3 Implications

AKI is commonly caused by factors such as sepsis, circulatory shock, cardiac surgery or other major surgeries, nephrotoxic drugs, and contrast agents (Kellum et al., 2013). These factors lead to oxidative stress, inflammation, cell death, and mitochondrial dysfunction in the kidneys (Gonsalez et al., 2019). The occurrence of AKI significantly affects patient prognosis; however, effective drugs for clinical treatment are still lacking (Moore et al., 2018). Many studies have demonstrated the pharmacological effects of quercetin, including antioxidant, anti-inflammatory, and anti-apoptotic properties (Hosseini et al., 2021; Chen Y. Q. et al., 2022; Di Petrillo et al., 2022). This study indicates that quercetin can reduce BUN and sCr levels, alleviate oxidative stress and inflammatory response, and improve renal injury scores in AKI models. Nevertheless, these results are based on a meta-analysis of preclinical animal experiments.

Animal experiments provide insights into disease mechanisms, explore potential treatment approaches, and investigate their feasibility and safety. However, animal experiments have inherent limitations: they may be susceptible to systematic bias and publication bias, and there are significant differences between animal studies and clinical practice, which pose challenges for translating findings into clinical applications (van der Worp et al., 2010; McGonigle and Ruggeri, 2014). Therefore, conducting meta-analyses and systematic reviews is necessary to further validate the reliability of animal experiment results and establish a foundation for clinical trials. Moreover, more well-designed animal experiments, large-scale randomized controlled trials, and cohort studies are needed to facilitate the clinical translation of our research.

### 4.4 Strengths and limitations

Initially, publication bias towards positive outcomes in animal studies raises the potential for an overestimation of quercetin’s efficacy. Additionally, the majority of included studies were conducted using standard animal models without considering the presence of other comorbidities. However, in a realistic clinical scenario, AKI patients often exhibit more complex conditions. Furthermore, the current pre-clinical investigations lack data from large animal models that closely resemble humans in terms of pathophysiological characteristics. These limitations significantly restrict the generalizability of our findings to human pathology.

## Data Availability

The original contributions presented in the study are included in the article/Supplementary Material, further inquiries can be directed to the corresponding authors.
